# Monocytic and neutrophilic CD11b and CD64 in severe sepsis

**DOI:** 10.1186/cc11728

**Published:** 2012-11-14

**Authors:** J Jämsä, V Huotari, ER Savolainen, H Syrjälä, T Ala-Kokko

**Affiliations:** 1 University of Oulu, Finland; 2 Oulu University Hospital, Oulu, Finland

## Background

Leukocyte immunophenotyping could improve sepsis diagnostics [1,2]. Our hypothesis was that monocytic and neutrophilic CD11b and CD64 antigen fluorescence intensities differ between severe sepsis, non-inflammatory ICU patients and nonseptic inflammation (off-pump coronary artery bypass (OPCABG)).

## Methods

Monocytic and neutrophilic CD11b and CD64 expressions were analyzed from 27 patients with severe sepsis, seven OPCABG patients and from eight ICU patients who did not fulfill any SIRS criteria. Blood samples were collected within 48 hours from the beginning of severe sepsis or in non-SIRS patients from ICU admission and two consecutive days (D0, D1, D2). From surgical patients, the first samples were taken on the day of surgery before the skin incision and two consecutive days (D0, D1, D2). In addition 10 healthy individuals served as controls. Samples were collected, processed and analyzed using flow cytometry as previously described [3].

## Results

**Figure 1:**
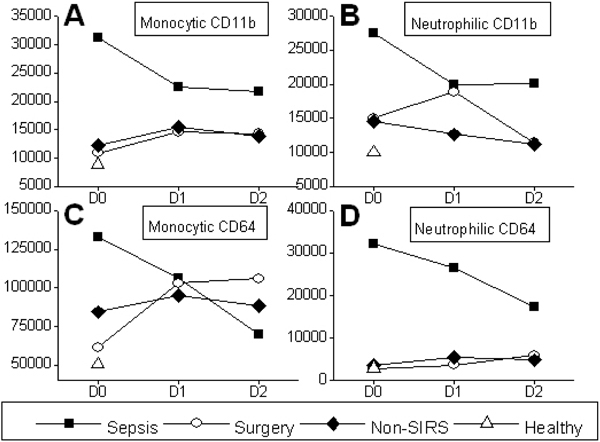
**Antigen fluorescence intensities in four groups in 3 days**.

The maximum fluorescence intensities of monocytic and neutrophilic CD11b and CD64 were highest in septic patients compared with the other groups (*P *< 0.05) (Figure 1). In severe sepsis, fluorescence intensities decreased over time (*P *< 0.05). In OPCABG the fluorescence intensities of other antigens increased from D0 to D1 except neutrophilic CD11b (*P *< 0.05). The intensities of other antigens except neutrophilic CD64 were lower in the healthy than in all the other groups (*P *< 0.05). Neutrophilic CD64, as well as other antigens, were lower in healthy controls compared with severe sepsis at all time points (*P *< 0.05).

## Conclusion

Based on this study, monocytic CD11b and neutrophilic CD64 could be helpful in distinguishing severe sepsis from nonseptic inflammation and healthy controls.
